# Data on tuning phase crystal of boron-doped manganese oxide and activation in removal of formaldehyde

**DOI:** 10.1016/j.dib.2024.111037

**Published:** 2024-10-16

**Authors:** Hoang-Huy Nguyen, Quoc-Tan Trinh, Quoc-Dat Le, Thanh-Thao Pham-Ngoc, Ngoc-Thien Nguyen, Thuy-Duong Nguyen-Phan, Cong-Danh Nguyen, Duy-Nhan Tran, Long Quang Nguyen, Dung Van Nguyen, Tuyet-Mai Tran-Thuy

**Affiliations:** a Faculty of Chemical Engineering, Ho Chi Minh City University of Technology (HCMUT), 268 Ly Thuong Kiet Street, District 10, Ho Chi Minh City, Vietnam; b Vietnam National University Ho Chi Minh City, Linh Trung Ward, Thu Duc City, Ho Chi Minh City, Vietnam

**Keywords:** B/OMS-2, Pyrolusite, Formaldehyde oxidation, Hydrothermal method

## Abstract

The data in this work provides the effect of aging temperature on manganese oxide allotropes prepared by hydrothermal method in the presence of boron dopant. The synthesized samples were labeled as B/Mn_x_O_y_ -100, B/Mn_x_O_y_ -150, and B/Mn_x_O_y_ -180 corresponding to the hydrothermal temperature at 100, 150, and 180 °C, respectively. X-ray diffraction (XRD) and inductively coupled plasma mass spectrometry (ICP-MS) methods were conducted to clarify the crystalline structure, as well as the amount of potassium, manganese, and boron elements in the three synthesized samples. Major cryptomelane crystal was achieved at 100 °C of aging temperature for B/Mn_x_O_y_ -100 sample. Pyrolusite which was detected as impurity phase over B/Mn_x_O_y_ -150 powder was characterized as main crystalline phase for B/Mn_x_O_y_ -180 sample. ICP-MS analysis proved an absence of potassium element only for the B/Mn_x_O_y_ -180 sample being in comprised of a push of potassium cations out the tunnel system of cryptomelane structure. Furthermore, scanning electron microscope (SEM) images evidenced noticeably morphological change from cryptomelane nanorods (for B/Mn_x_O_y_ -100) to pyrolusite slabs (for B/Mn_x_O_y_ -180) while increasing the aging hydrothermal condition from 100 to 180 °C. Size distribution diagrams were defined with the assistance of ImageJ and were plotted in Origin software. The diameter of nanorods were 14.8 ± 0.3 nm for B/Mn_x_O_y_ -100 and 17.2 ± 0.4 nm for B/Mn_x_O_y_ -150 while that was 100.3 ± 2.5 nm for B/Mn_x_O_y_ -180 sample indicating an aggregation of nanorods into slabs at 180 °C of aging temperature. Formaldehyde conversion data were collected and computed with a gas chromatograph flame ionization detector (GC-FID) instrument. The B/Mn_x_O_y_ -180 material deteriorated into formaldehyde degradation at 80 °C of oxidation reaction temperature suggesting an inactivated pyrolusite phase for catalytic oxidation. The formaldehyde removal efficiency over the B/Mn_x_O_y_ -100 material reached 28.4 ± 1.2 % and 36.9 ± 0.9 % at 80 and 100 °C, respectively offering an active cryptomelane-phase of B/Mn_x_O_y_ -100 for formaldehyde abatement at low reaction temperature. Altogether, the current data provided valuable insights into the influence of aging temperature on the crystalline of manganese oxide-based materials and a feasibly catalytic performance of B/Mn_x_O_y_ -100 cryptomelane in formaldehyde elimination.

## Graphical abstract

**Figure:**
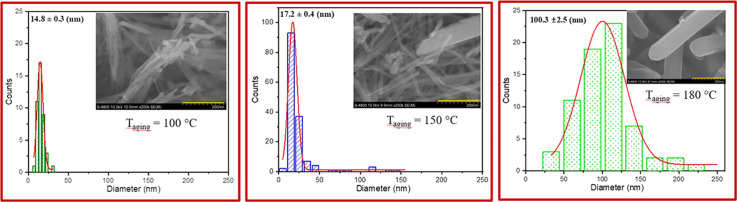

Specifications Table

**Table:** 

Subject	Materials chemistry
Specific subject area	Catalysis, Environmental remediation
Type of data	Tables, Figures
Data collection	The synthesized materials were characterized via various methods. XRD was employed on Bucker AXS D8 to determine crystal phases of manganese oxides and was plotted Origin software. Chemical composition was analyzed on a OptimaTM 8000 ICP-OES. SEM images were captured on a Hitachi S4800, and diameter of rods were measured by using ImageJ software and diagrams of size distributions were plotted in Origin. The catalytic activity was evaluated in deep oxidation of formaldehyde, and formaldehyde conversion was validated by GC-FID.
Data source location	Ho Chi Minh City University of Technology (HCMUT), Ho Chi Minh City, Vietnam
Data accessibility	Repository name: Mendeley Data Data identification number: 10.17632/r48x4bmx7g.2 Direct URL to data: https://data.mendeley.com/datasets/r48x4bmx7g/2
Related research article	None

## 1 Value of the Data

• These data are useful in understanding how thermal treatment alters the physi-chemical properties and the crystalline phases of the manganese oxide materials.
• The data about formaldehyde oxidation ability of boron-modified cryptomelane is useful for future optimization and applications.
• Researchers working in materials science, specifically materials for environment remediation, can benefit from the data in this article.

## 2 Background

Allotropes of crystalline manganese oxides as cryptomelane (α- MnO_2_), pyrolusite (β- MnO_2_) and ramsdellite (γ- MnO_2_) have been concerned because of different performances in electrical energy storage field, sustainable energy, catalytic oxidation and environmental preservation. Cryptomelane, another well-known name for α- MnO_2_, possesses 2×2 tunnel structure with pore size of 4.6 Å [1], the average manganese oxidation state of 3.6 [2], and potassium ions residing in the grooves to maintain the framework's charge balance [2,3]. Furthermore, crystal cryptomelane structure could be modified by conducting metallic and non-metallic dopants leading a significant enhancement in defective oxygen species. These promoted the modified cryptomelane materials in variously potential applications in environmental remediation [4], green catalysis and effectively catalytic activity in deep oxidation of volatile organic compounds [5]. The current data provide the effect of hydrothermal conditions on structural polymorphs of boron-doped manganese oxide. A preliminary test of catalytic performance over the synthesized manganese oxides in deep oxidation of formaldehyde deserves further optimization.

## 3 Data Description

This data section provides the effect of aging temperature on allotropes of synthesized manganese oxide by conducting the hydrothermal method with the aid of non-metallic boron in the precursor. The hydrothermal aging temperature was investigated at 100, 150 and 180 °C and the obtained samples were denoted B/Mn_x_O_y_ −100, B/Mn_x_O_y_ −150 and B/Mn_x_O_y_ −180 materials, respectively. Moreover, a preliminarily catalytic performance testing in deep oxidation of formaldehyde were conducted to grasp the feasibility of these material in removal of air pollution.

**Fig. 1:**
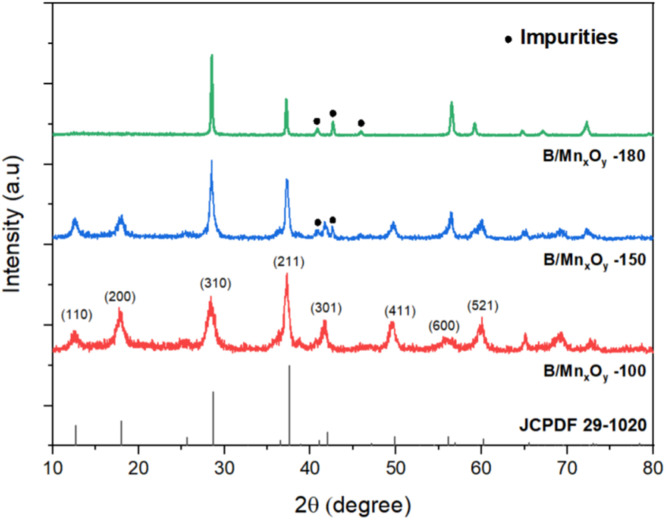
XRD patterns of B/Mn_x_O_y_ samples at different aging temperature treatments.

XRD patterns in Fig. 1, present the diffraction peaks of the synthesized materials prepared in various autoclave temperature treatments. At 100 °C of reaction temperatures, XRD patterns of B/Mn_x_O_y_ −100 sample shows clearly diffraction peaks at 2θ of ∼12.8°, 18.1°, 28.7°, 37.6°, 42.0°, 49.9°, 56.2°, and 60.2° consisting with the (110), (200), (310), (211), (301), (411), (600) and (521) crystal planes of cryptomelane (JCPDS-29–1020) [6]. It reveals that the presence of boron in the precursor does not destroy the crystalline cryptomelane structure for the B/Mn_x_O_y_ −100 material. The increase of autoclave reaction temperature to 150 °C leads to the appearance of some new diffraction peaks with low intensity at 2θ of ∼40.8°, and 42.7°; along with the main diffraction reflectance angles attributed to the cryptomelane structure. At 180 °C of autoclave reaction temperature, the three cryptomelane-crystal planes of (110), (200), and (411) vanish and the three angles of diffraction 2θ at ∼40.8°, 42.7°, and 46° become more significant. This indicates that high temperature of hydrothermal treatment facilitates phase transition from crystal cryptomelane (for B/Mn_x_O_y_ -100) to the pyrolusite crystalline of β-MnO_2_ (for B/Mn_x_O_y_ -180).

Table 1 shows the elemental composition of B/Mn_x_O_y_ -100, B/Mn_x_O_y_ -150 and B/Mn_x_O_y_ -180 samples defined from ICP-MS analysis. All of the three samples present ∼0.1 wt% of boron loading and the amount of manganese is in the range of 53–57 wt%. The small boron dopant amount for the current boron-doped cryptomelane materials is comparable to other reported sample of 0.12 wt% B-OMS-2 [7]. It proves that the boron is hard to dope in the cryptomelane structure, no matter what the method is. Noticeably, the potassium content is 3.76 wt% for B/Mn_x_O_y_ -100 sample, reduced to 3.32 wt% for B/Mn_x_O_y_ -150 material and that vanishes for B/Mn_x_O_y_ -180 one. It was also reported that potassium ions were absent in the synthesized pyrolusite material, though the potassium element was one of important species in the potassium permanganate precursor [8]. We, therefore, assumed that when the (2 × 2) structure of OMS-2 changed to the (1 × 1) pyrolusite structure, the groove size significantly decreased causing a push of potassium cations out of cryptomelane tunnels. This consists of a lack of potassium elements in the current pyrolusite structure of synthesized B/Mn_x_O_y_ -180 material. Furthermore, other work also reported a major phase of crystalline pyrolusite could be prepared by oxidation of precursor manganese sulfate with potassium chlorate at 180 °C [9]. The current achieved results prove a phase transformation from cryptomelane to pyrolusite under elevation of aging temperature from 100 to 180 °C in preparation of boron-doped manganese oxides.

**Table 1:** Elemental composition achieved from the ICP-MS analysis.

Sample name	wt.% K	wt.% Mn	wt.% B
B/Mn_x_O_y_ -100	3.76	53.25	0.11
B/Mn_x_O_y_ -150	3.32	57.08	0.11
B/Mn_x_O_y_ -180	0	56.70	0.10

**Fig. 2:**
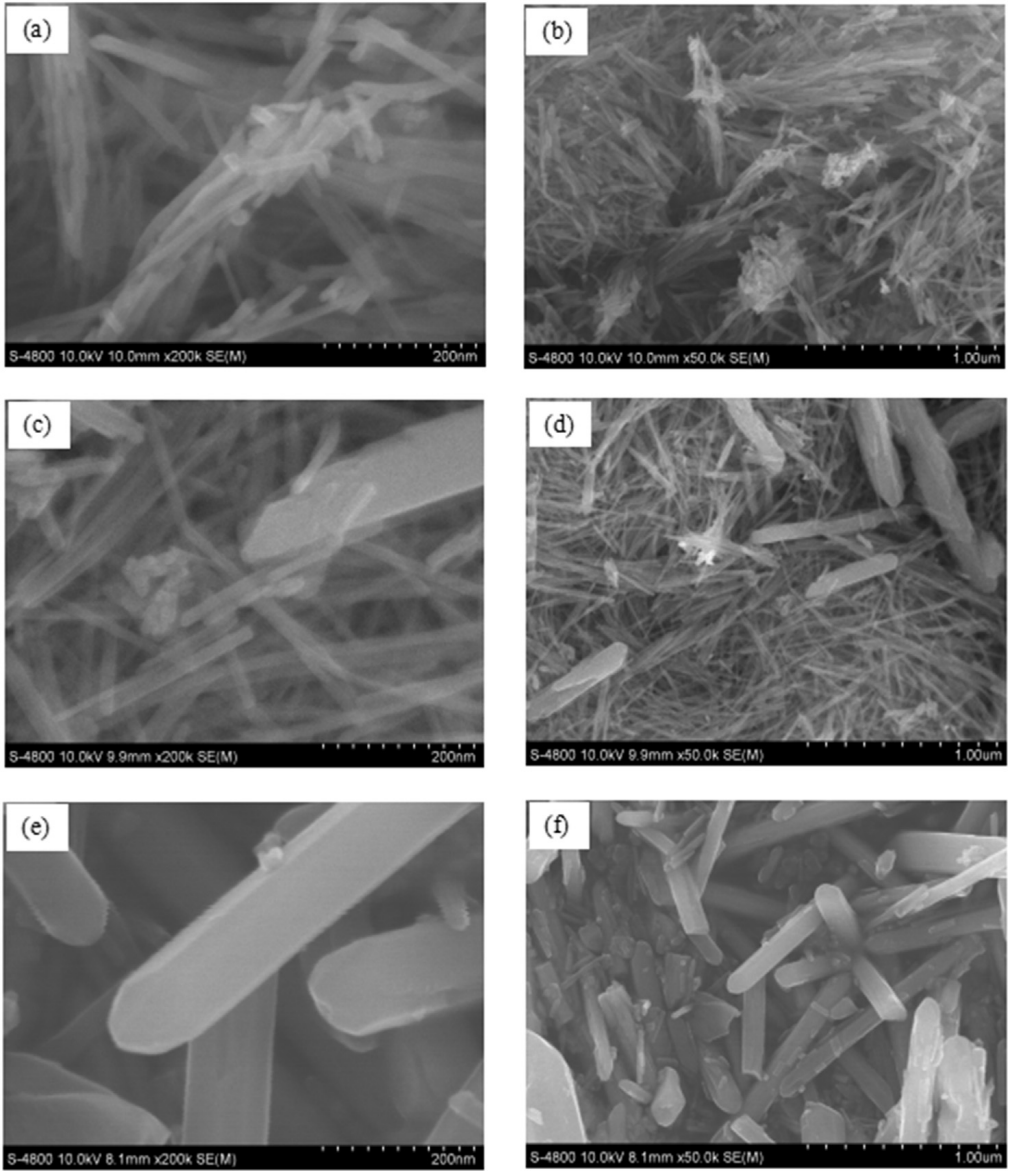
SEM images of boron-doped cryptomelane samples: (a), (b): B/Mn_x_O_y_ -100; (c), (d): B/Mn_x_O_y_ -150, (e), (f): B/Mn_x_O_y_ -180.

**Fig. 3:**
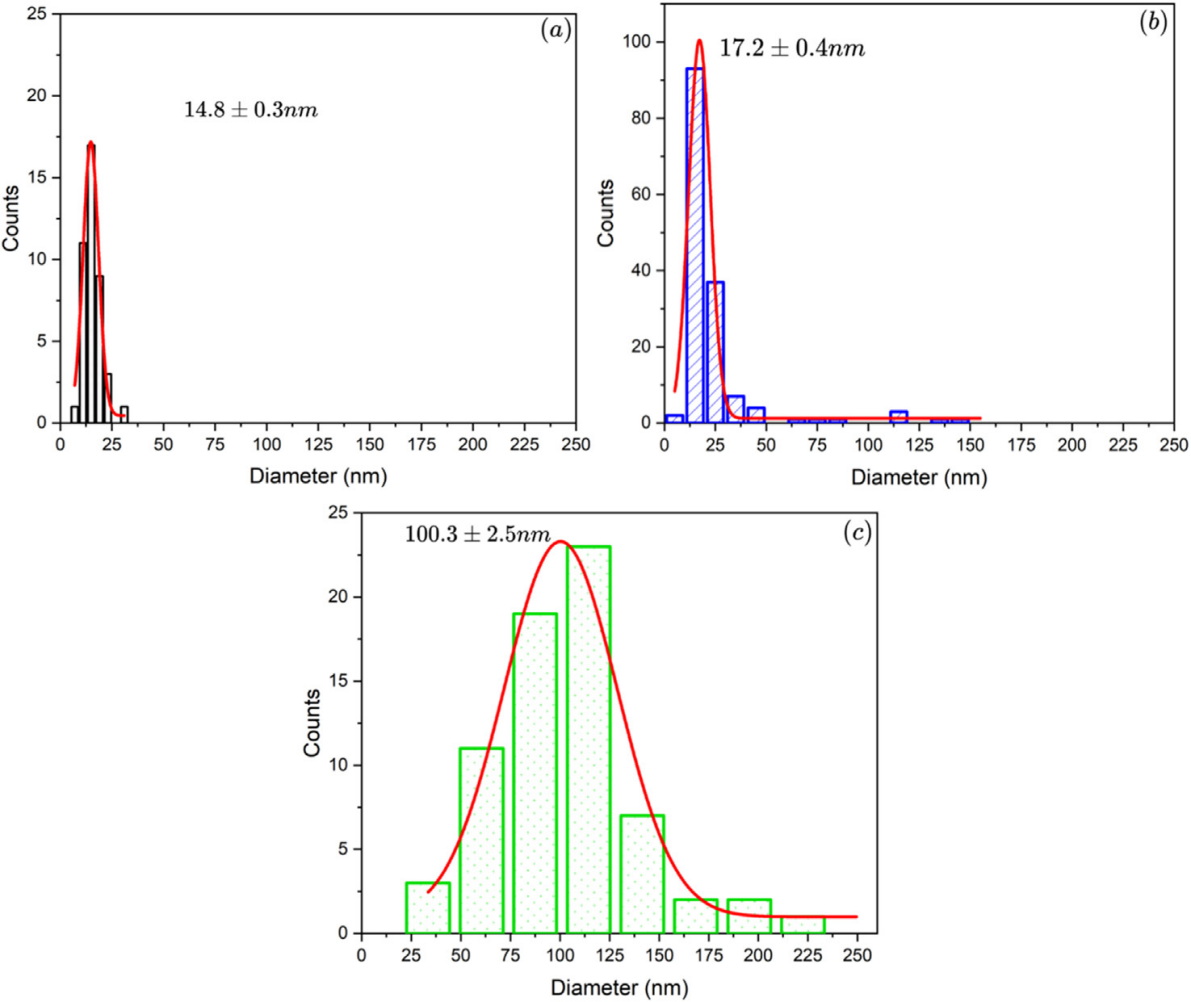
Size distribution of (a) B/Mn_x_O_y_ -100; (b) B/Mn_x_O_y_ -150; (c) B/Mn_x_O_y_ -180.

The morphologies of boron-doped cryptomelane samples collected from SEM images are presented in Fig. 2. It can be noticed that the B/Mn_x_O_y_ -100 material has a rod-shaped structure and that such morphology resembles other report of cryptomelane prepared by reflux approach [3]. The B/Mn_x_O_y_ -180 sample exhibits a majority of large-formed slabs/bars and a minority of nanorods obtained from the SEM images (Fig. 2e, and F). However, only several bars could be detected over the B/Mn_x_O_y_ -150 sample, in company with the most of nanorods (Fig. 2c, and d). The size distributions of synthesized materials (Fig. 3) reveal an incline of diameter with increasing autoclave reaction temperature. The diameter of nanorods grows slightly from ∼15 nm to ∼17 nm while increasing the aging reaction temperature from 100 to 150 °C (Fig. 3a, and b). Furthermore, an agglomeration of nanorods to slabs with bigger size of ∼100 nm diameter over B/Mn_x_O_y_ -180 is recognized (Fig. 3c) providing an essential effect of aging temperature on morphological alternation and crystalline transformation for manganese oxide materials.

**Fig. 4:**
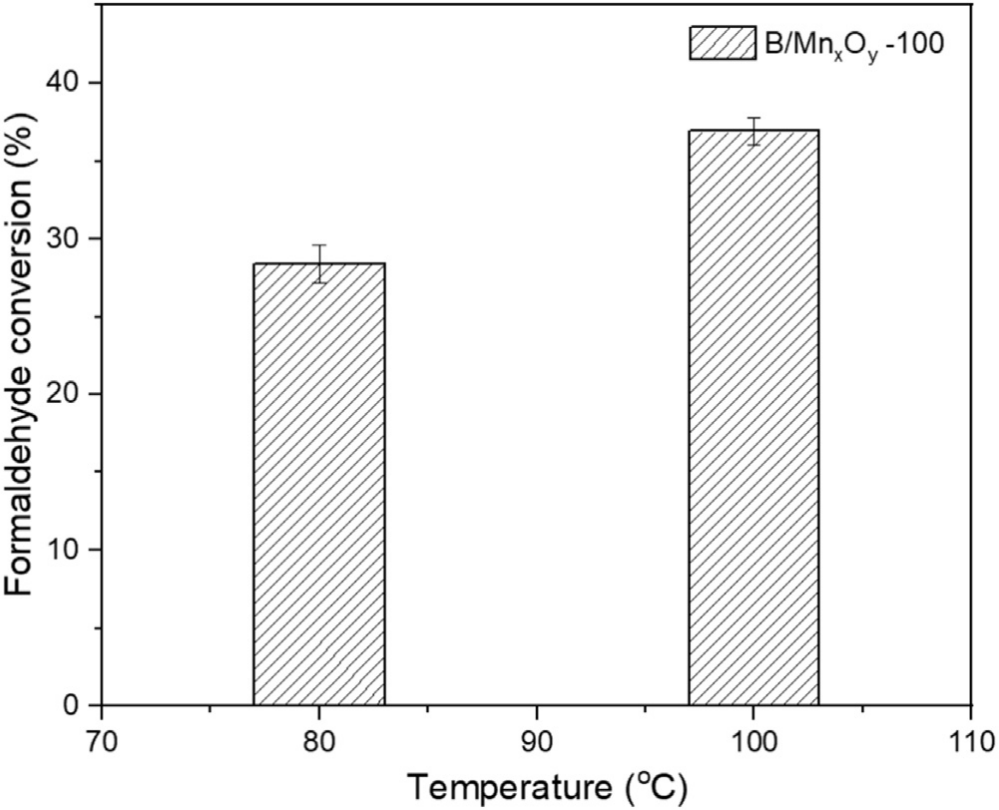
The formaldehyde conversion chart with B/Mn_x_O_y_-100 catalyst.

Fig. 4 shows the catalytic activity in deep oxidation of formaldehyde over manufactured B/Mn_x_O_y_ samples at 80 and 100 °C of oxidation reaction temperatures. As the reaction temperature rises, B/Mn_x_O_y_ -100 sample presents an incline of formaldehyde conversion. The formaldehyde removal efficiency over the B/Mn_x_O_y_ -100 catalyst reaches 28.4 ± 1.2 % at 80 °C and gets 36.9 ± 0.9 % at 100 °C of the oxidation temperature. For B/Mn_x_O_y_ -150 and B/Mn_x_O_y_ -180 catalysts, the performance is disappointingly low, with the formaldehyde removal efficiency of about 0 % over B/Mn_x_O_y_ -180, at 80 °C of oxidation temperature. This suggests a beneficial material of cryptomelane-based B/Mn_x_O_y_ -100 in facilitation of formaldehyde-deep oxidation at low reaction temperatures (*T* < 100 °C). Pyrolusite decays, otherwise, catalytic ability in oxidation abatement of formaldehyde at 80 °C.

## 4 Experimental Design, Materials and Methods

### 4.1 Chemicals

Potassium permanganate (KMnO_4_, 99.5 % purity), manganese sulfate monohydrate (MnSO_4_.H_2_O, 99 % purity), boric acid (H_3_BO_3_, > 99.5 % purity), and nitric acid (HNO_3_, 65–68 % purity) were originated from China. Distilled water was obtained from the Catalysis Laboratory and Physical Chemistry Laboratory, Department of Chemical Engineering, University of Technology, Ho Chi Minh City.

### 4.2 Synthesis of materials

Potassium permanganate (KMnO_4_) was typically dissolved in 100 mL of distilled water in an Erlenmeyer flask to make solution A. Meanwhile, manganese sulfate monohydrate (MnSO_4_.H_2_O) was dissolved in 30 mL of distilled water in a 100 mL beaker and then boric acid (H_3_BO_3_) was added to acquire solution B. Pouring solution B to solution A to obtain final mixture, which molar concentrations of KMnO_4_, MnSO_4_ and H_3_BO_3_ in the reactant mixture were 0.3 M, and 0.4 M and 0.02 M, respectively. The pH <1 was further adjusted by adding concentrated nitric acid [3] and was stirred for 30 min at room temperature before aging in an autoclave for 24 h at different hydrothermal temperatures. After the hydrothermal process, the sample was washed, filtrated, and dried at 80 °C overnight. The product was finely ground and collected. In this work, three as-prepared samples were aged at autoclave treatment temperatures of 100 °C, 150 °C, and 180 °C. The synthesized samples were denoted as B/Mn_x_O_y_ −100, B/Mn_x_O_y_ −150, and B/Mn_x_O_y_ −180, respectively.

### 4.3 Characterization

X-ray diffraction (XRD, Bucker AXS D8) was utilized to determine the crystal structure of the material. Chemical composition was examined by using inductively coupled plasma-mass spectrometry (ICP-MS, OptimaTM 8000 ICP-OES). A scanning electron microscope (SEM) was conducted to record the images of the synthesized sample by utilizing a Hitachi S4800.

### 4.4 Catalytic activity testing

The catalytic activity of synthesized B/Mn_x_O_y_ for removal was investigated by using a fixed-bed reactor wherein a 0.1 g catalyst was set between two layers of glass wool. The reactant mixture containing nitrogen, oxygen, and formaldehyde vapor flows was prepared. Formaldehyde vapor was formed by conducting nitrogen flow through the 37 % formaldehyde solution maintained at 0 °C. A 50 mL total reactant flow was set with 90 ppm of the concentration of formaldehyde. The inlet and outlet of formaldehyde concentration were analyzed by gas chromatography with flame ionization detection system. Formaldehyde conversion was determined by the following equation:

$$H\;\left(\%\right)\;=\;\frac{{\left[\text{HCHO}\right]}_{\text{in}}-{\left[\text{HCHO}\right]}_{\text{out}}}{{\left[\text{HCHO}\right]}_{\text{in}}}\times 100\;\%$$

wherein [HCHO]_in_ and [HCHO]_out_ were characterized as inlet and outlet of formaldehyde concentrations.

## Limitations

Not applicable

## Ethics Statement

There are no animal tests, human subjects, or data gathered from social media platforms in the current work.

## CRediT authorship contribution statement

**Hoang-Huy Nguyen:** Visualization, Formal analysis, Writing – original draft. **Quoc-Tan Trinh:** Visualization, Formal analysis, Writing – original draft. **Quoc-Dat Le:** Visualization, Formal analysis, Writing – original draft. **Thanh-Thao Pham-Ngoc:** Visualization, Formal analysis, Writing – original draft. **Ngoc-Thien Nguyen:** Investigation. **Thuy-Duong Nguyen-Phan:** Investigation. **Cong-Danh Nguyen:** Investigation. **Duy-Nhan Tran:** Investigation, Writing – original draft. **Long Quang Nguyen:** Methodology, Resources. **Dung Van Nguyen:** Methodology. **Tuyet-Mai Tran-Thuy:** Conceptualization, Methodology, Supervision, Writing – review & editing.

## Data Availability

Mendeley DataData on tuning phase crystal of boron-doped manganese oxide and activation in removal of formaldehyde (Original data).
